# A prospective cohort study of Cutaneous Leishmaniasis due to *Leishmania major*: Dynamics of the Leishmanin skin test and its predictive value for protection against infection and disease

**DOI:** 10.1371/journal.pntd.0008550

**Published:** 2020-08-25

**Authors:** Jihène Bettaieb, Amine Toumi, Wissem Ghawar, Sadok Chlif, Mariem Nouira, Nabil Belhaj-Hamida, Adel Gharbi, Nissaf Ben-Alaya, Dhafer Laouini, Hechmi Louzir, Koussay Dellagi, Afif Ben Salah

**Affiliations:** 1 Department of Medical Epidemiology, Institut Pasteur de Tunis, University of Tunis El Manar, Tunis, Tunisia; 2 Laboratory of Transmission, Control and Immunobiology of Infections (LR11IPT02), Institut Pasteur de Tunis, University of Tunis El Manar, Tunis, Tunisia; 3 Department of Epidemiology, Observatoire National des Maladies Nouvelles et Emergentes, Tunis, Tunisia; 4 Department of Family and Community Medicine, College of Medicine and Medical Sciences, Arabian Gulf University, Manama, Bahrain; University of Notre Dame, UNITED STATES

## Abstract

**Background:**

Leishmanin Skin Test (LST) is considered as a useful indicator of past infection by *Leishmania* parasites. However, the temporal dynamics of a positive LST under different epidemiologic scenarios and whether it relates to the protection against the recurrence of an overt disease are not fully documented.

**Methodology/Principal findings:**

We report here on a population based prospective study conducted on 2686 individuals living in two foci located in Central Tunisia, to assess over a one-year epidemiologic season, the incidence of *Leishmania* (*L*.) *major* infection and disease and changes in LST reactivity. The two foci were both endemic for Cutaneous Leishmaniasis (CL) due to *L*. *major*, but contrasted in their history for this disease (ie: an old focus versus a recent focus).

We found that most infections occurred in the new focus (290/1000; 95% CI: 265–315 person-years) with an incidence rate of CL lesions 2.4 times higher than in the old focus. Likewise, the rates of LST reactivity reversion and loss, in the new focus, were 99/1000[38–116] person-years and 14/1000[8–21] person-years, respectively. Loss of LST reactivity was not noticed in the old focus. Interestingly, the incidence rates of symptomatic infection did not differ significantly according to the LST status at enrolment (negative versus positive) between the combined foci and the new one.

**Conclusions/Significance:**

Our findings confirm LST as a good tool for assessing *L*. *major* cryptic infection. However, the instability of the LST positivity in new foci should be considered as an important confounder of the outcome of this infection when developing a research protocol for vaccine trial.

## Author summary

This population-based cohort study is the first one conducted in the Old World which compares the incidence of Zoonotic Cutaneous Leishmaniasis (ZCL) to the temporal dynamics of LST in two foci contrasting in their past history for *L major* transmission. Our results demonstrate that people living in an old CL focus with intense and sustained exposure to *L major*, develop long-lasting immunity against the parasite resulting in protection against overt disease; This feature markedly differs from that observed in more recent CL focus where overt CL occurs regardless of LST status. Our study highlights that although LST is a useful epidemiological tool for monitoring leishmania transmission rate over time, its use as a surrogate marker of protection against recurrence of CL or as an inclusion criterion in vaccine trials, calls caution. Moreover, the intensity and past history of *Leishmania* transmission in the study site should be anticipated as important confounders of the outcome of infection and should be carefully addressed in the design of anti-*Leishmania* vaccine trials.

## Introduction

Leishmaniasis is a group of vector-borne diseases transmitted by the bite of phlebotomine sand-flies that generate a heavy disease burden at the global level. In Tunisia (North Africa), the yearly incidence of Cutaneous Leishmaniasis (CL) is ~20 to 30 per 100,000 persons. The most frequent form, known as Zoonotic Cutaneous Leishmaniasis (ZCL), is caused by *Leishmania* (*L*.) *major* with rodents as animal reservoir and occurs in Central and Southern Tunisia [1]. The parasite is transmitted to humans during summer through the bite of infected female sandflies; with active cutaneous lesions emerging during autumn and winter and evolving until spring. Hence the epidemiologic season of ZCL extends over one year from May to May of the next year. The Leishmanin Skin Test (LST) or Montonegro test, is the most reliable and easy to perform assay to assess in field studies, past or present infection of exposed individuals by *Leishmania spp* [2, 3]. Therefore, in epidemiological surveys, LST positivity in individuals without active cutaneous lesions or typical scars or any history of patent cutaneous sore (s), is considered as indicative of prior asymptomatic infection [4]. It is well known that patients developing overt ZCL are to some degree, protected against disease recurrence when exposed in subsequent years to infected sand fly bites; Hence, LST positivity is inferred to correlate with this protection [4, 5] and LST negative individuals are considered as constituting the pool of susceptible in the exposed population. We have epidemiological evidence that the protection conferred by past *Leishmania* infection is neither lifelong nor absolute, and that resistance to reinfection is lost with time, particularly in the absence of continuous boosting of *Leishmania* specific immune responses by infectious sandfly bites [5]. Furthermore, field observations for decades in Tunisia, strongly suggest that clinical features and severity of ZCL are highly influenced by the age of parasite transmission in the area where the exposed population is living (ie: old focus with long lasting transmission versus young or recent focus where *L major* transmission was only recently recorded).

The main purpose of this prospective cohort study conducted in Central Tunisia was to measure, over a complete ZCL transmission cycle (one year), changes in LST reactivity and monitor *L*. *major* infection and disease, under two different epidemiologic scenarios: an old ZCL focus compared to a recent (young) one. Evaluation of these parameters is critical for understanding the dynamic of ZCL transmission patterns and for providing evidence basis for control measures and design of vaccine trials.

## Methods

### Fieldwork

A prospective cohort study was simultaneously conducted from March 2009 to May 2010 in five villages endemic for ZCL, located in the Governorates of Sidi-Bouzid (Mbarkia, Dhouibet) and Kairouan (Mnara, Msaadia, Ksour), Central Tunisia. According to the local surveillance system, Mnara has evidence of a long-established *Leishmania* transmission in the region (cases have been reported since 1981), while the 4 remaining villages are considered as new foci (cases have been reported since 2005 ie: less than 5 years before the implementation of the present study).

### Enrolment and follow-up of participants

This study was performed under the international (Declaration of Helsinki) and national regulations. The study protocol was reviewed and approved by the ethical committee of Institut Pasteur de Tunis as well as the Ministry of Health and its regional representatives in Central Tunisia.

The cohort was based on households that were selected from each village through a two-stage cluster sampling scheme with clusters of equal sizes based on an updated census list provided by the Tunisian National Institute of Statistics. The first stage consisted of a random selection of 25 districts (each district contains about 70 dwellings in general) from the five villages. The second stage consisted of a random selection of ~25 to 30 households per district to permit a sub sample of ~75 to 100 volunteers per district.

At each sampled household, every resident aged 5 to 65 years was enrolled in the cohort after written informed consent obtained from each participant and/or their parents/legal guardians, as appropriate.

At baseline (March-May 2009), just before start of the *L*. *major* transmission season, information was collected on household members with regard to demographic (age, gender), socio-economic (education, annual income, occupation), past family and personal history of CL, and description of the dwelling. All eligible individuals were physically examined for detection of typical scars or lesions and were tested using LST (LST1).

LST was performed by intradermal injection of 100 microliters of Leishmanin suspension containing 5x10^6^/ml of *L*. *major* promastigotes in 0.5% phenol saline. The induration was measured along two diameters by the ball point pen technique after 48h by the Sokal’s technique [6]. Induration with a diameter of 5 mm was considered as a positive response. The skin test antigen used was prepared and provided by Pasteur Institute of Iran **(**reference strain MRHO/IR/75/ER). Preparation and quality control of Leishmanin were performed according to a previously published protocol [7]. Briefly, promastigotes of *L*. *major* were grown in NNN medium, then transferred to RPMI 1640 medium supplemented with 40% fetal calf serum (FCS) and incubated at 25°C. Cultured promastigotes were harvested at stationary growth phase, centrifuged at 4000 g for 30 minutes and washed with saline solution.

To allow an early detection of incident ZCL cases in the study population, every enrolled dwelling was visited twice a month, during nine months (from September 2009 to May 2010) all over the season of disease emergence. All individuals who developed lesions were parasitologically confirmed: parasites were detected i) in exudates collected from lesions smeared on slides and ii) in culture using coagulated rabbit serum (CRS) medium [8]. All ZCL cases were referred to the nearest health center for case management.

A second LST was applied to all volunteers on May 2010, just before the next transmission season (LST2). A stringent quality control was applied to the LST procedures to reduce the risk of systematic errors [9]. The two tests were performed for the same patient by the same technician according to a standard protocol, so as to avoid common causes of variation in the execution (the amount of injected antigen; the site and depth of the injection and the physiological status of the patient). The reaction of each subject was read two times by each of two different observers under blind conditions as much as possible.

### Outcomes definitions and analysis plan

All individuals of the cohort were followed as described during one full epidemiologic season (12 months) to detect and investigate those who developed the disease. This information permitted to assess the following incidence densities:

$$\frac{Number\;of\;cases\;onset}{Person\;time\;at\;risk\;(PTR)}$$

- rate of LST conversion (incidence of infection):
  Number of persons who converted their reaction (a negative LST1 (LST size < 5 mm) and a positive LST2 (LST > = 5 mm)] / (PTR),
- rate of LST reversion:
  Number of persons with positive LST1 and negative LST2 / PTR,
- rate of LST reactivity loss:
  Number of persons with LST reversion in which there was at least a 5 mm decrease in the mean reaction size of LST2 compared to LST1/ PTR,
- incidence of Leishmaniasis cases (symptomatic infection):
  Number of persons without any active skin lesion at enrolment that later develop an active lesion diagnosed as leishmaniasis/ PTR.

Person-time at risk (PTR) was calculated differently for infection and disease rates, because persons were regularly under surveillance for disease but assessed only two times for infection: at enrolment before the ZCL transmission season, and at the end of follow up, one year later just before the next transmission season. As for disease, a subject is eligible to contribute person-time to the study as long as that person is disease-free i.e. still remains at risk of developing the first lesion of interest. As for infection, the time contributed by each subject at risk was assumed to be one year.

Rates of LST conversion, reversion, and loss of LST reactivity were measured only on the pool of persons who were tested twice (i.e. for LST1 and LST2). To contribute to person-time at risk, participants were required to be LST1 negative for LST conversion, and LST1 positive for reversion and for loss of LST reactivity.

The LST reactivity at enrolment was considered to refine the estimation of the incidence of leishmaniasis cases according to LST result at baseline.

Rates with 95% Confidence Interval (CI) were calculated for both “focus category” and “alternative focus category” and stratified by age groups and gender. The Relative Risk (RR) of developing a CL lesion was also calculated for each risk factor (focus, age and gender).

The sampling design was taken into account in the analysis. Data were weighted to reduce the potential for bias due to nonresponse. Weights were calculated by means of the French INSEE software CALMAR 2 [10]. Raking ratio calibration[11] option was applied using age and gender distribution of the target population reported in the census data.

Between-group comparisons were carried out using T-tests, analysis of variance (ANOVA), or Mann-Witney test for continuous variables, and χ^2^ test or fisher's exact test for variables expressed in frequencies. A 5% significance level was adopted (p≤0.05).

Data were analyzed using STATA/IC 11.0 (StataCorp, College Station, TX).

## Results

A house to house survey recruited 2686 individuals with 2186 and 500 individuals living in the new and old focus respectively. LST1 was done and read in 1688 and 405 individuals in either focus, respectively. Most missing individuals were absent during household visits. Table 1 shows the basic characteristics of the study population.

**Table 1:** Selected baseline characteristics of study participants by focus (counts reflect weighting).

	Old focus (n = 405)	New focus (n = 1688)	Both foci (n = 2093)	P value
**Median Age (IQR**^1^**) (yrs)**	25 (14–43)	24 (14–38)	25 (14–39)	NS^3^
**Females % (95% CI**^2^**)**	54 (50–57)	52 (50–54)	52 (51–54)	NS
**Education % (95% CI)**				NS
Under school age	2 (0–3)	4 (3–5)	3 (2–4)	
Illiterate	24 (20–28)	21 (19–23)	21 (19–23)	
Primary	47 (42–52)	53 (51–56)	52 (50–54)	
Secondary	26 (22–30)	21 (19–23)	22 (20–24)	
University	2 (1–4)	2 (1–3)	2 (1–3)	
**Farming occupation % (95% CI)**	1 (0–3)	3(2–4)	3 (2–3)	NS
**History of ZCL/Scars % (95% CI)**	36 (33–39)	18 (17–20)	22 (21–24)	<10^−3^

^1^IQR: Interquartile range.

^2^CI: Confidence Interval.

^3^NS: Non significant

The weighted LST1 results showed that 56% of the population under study have been exposed to *Leishmania* parasites but with salient differences between the two foci: LST1 positivity was 2.3 times greater in the old focus (99%) than in the new focus (43%); (p<10^−3^).

**Fig 1:**
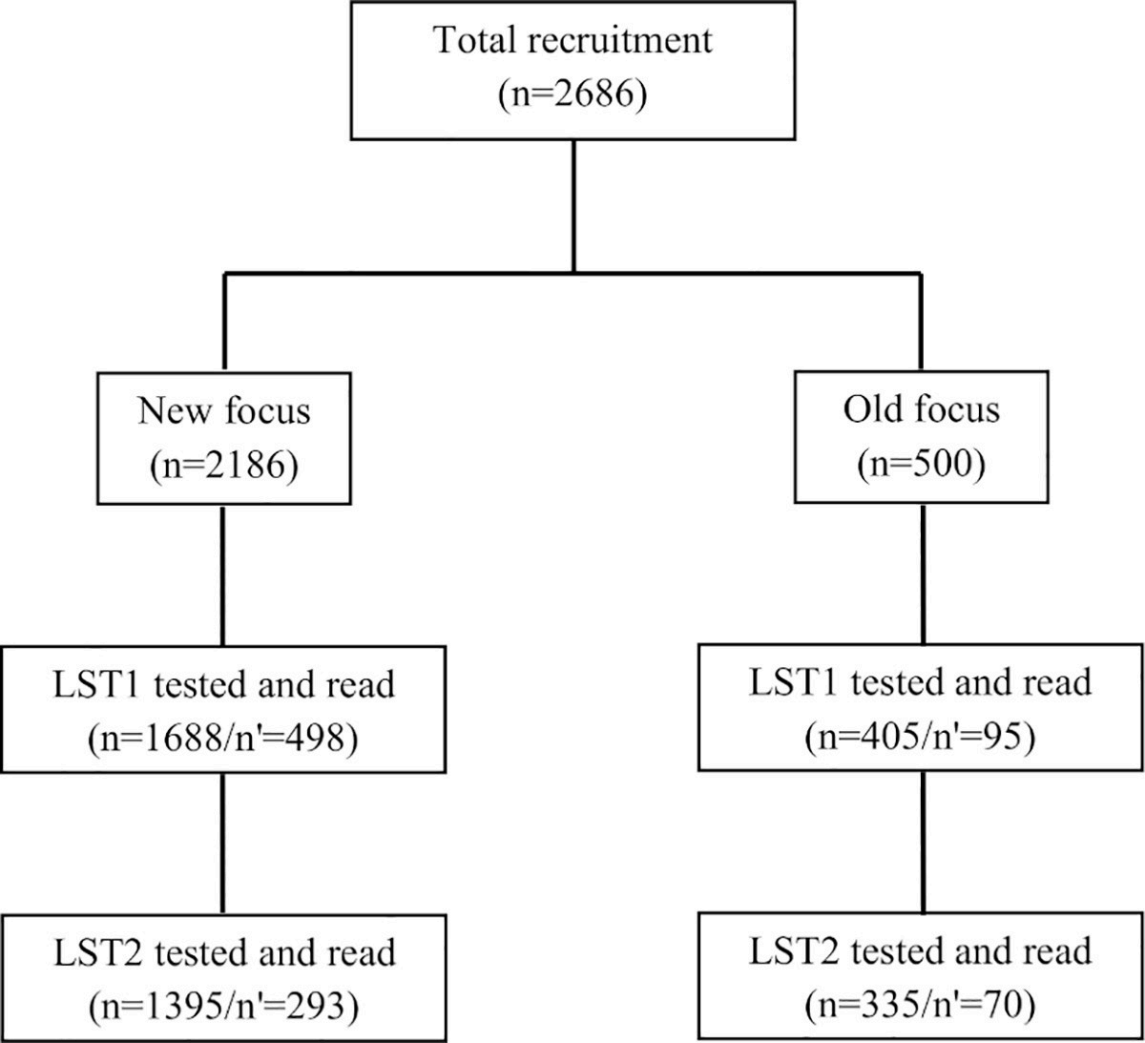
Flowchart of study participants enrolled and Leishmanin skin tested. LST: Leishmanin skin test, n: analyzed, n’: lost to follow up. LST1: The first LST performed at baseline: March-May 2009, just before *Leishmania major* transmission season. LST2: The second LST applied to all volunteers on May 2010, just before the next transmission season.

Between the first and the second LST, 363 subjects were lost from follow-up: one case died, 115 declined receiving LST2, and 247 others were not present in the dwelling during LST2 visits (17.3%). Hence, only 1395 (82.6%) individuals from the new focus and 335 (82.7%) from the old one were re-tested before the start of the next transmission season (Fig 1). The LST2 participation rates were almost similar in both areas. Except for personal history of LCZ, no statistically significant difference was found in the distribution by age, sex, educational level or agricultural activity between the two foci in the pool of LST1 and LST2 tested persons (Table in S1 Table). However, there was a significant difference between LST2 participants and non-participants with regard to sex and age. In fact, women, children and elderly were more likely to be at home at the time of the interviewer's visit than adult men.

### Dynamics of LST reactivity after one ZCL transmission season

#### LST conversion (Incidence of infection)

Among the 1730 LST participants tested before and after one-year epidemiologic season, 218 were LST1 negative at baseline but converted to LST2 positive and 98% among them lived in the new focus.

The incidence rate of infection (defined as LST conversion rate) was 295/1000 person-years at risk overall, and 290/1000 person-years in the new focus where most individuals LST negative at baseline were living (Table 2). In the old focus, the number of LST negative was so low at baseline as to allow any meaningful comparison (it should be calculated as 1000/1000 person-years). Likewise, the mean increase in LST induration size was higher in the old focus compared to the new one (10.5 mm [8.3–12.7] versus 8.8 mm [8.3–9.4]). Overall, asymptomatic infection rate, as measured by skin test conversion in the absence of any detectable lesion was 286/1000 [261–311] person-years. It was 281/1000 [256–306] person-years in the new focus. The very few individuals (four subjects) from the old focus who were LST1 negative at baseline all have converted to positive following LST2 without developing lesions. The incidence of infection was higher in older age (p <10^−3^), but did not significantly differ according to gender.

**Table 2:** Dynamics of Leishmanin Skin Test (LST) reactivity after one epidemiological season by focus, age and gender (Counts reflect weighting).

	Rate^1^ of LST conversion [95% CI^2^]			Rate^1^ of LST reversion [95% CI]		
				Any reversion		Reversion with loss
Focus	New	Old	Both	Old	New	New
**Total**	290 (265–315)	1000 (770–1000)	295 (270–321)	0	99 (83–116)	14 (8–21)
**Age (years)**						
[5–15[	207 (174–246)	-	210 (170–250)	0	90 (63–128)	17 (7–39)
[15–25[	147 (116–184)	1000 (646–1000)	160 (130–200)	0	68 (44–104)	22 (10–46)
[25–40[	414 (372–456)	1000 (601–1000)	420 (380–460)	0	126 (93–167)	10 (3–28)
[40–65]	397 (347–449)	-	400 (350–450)	0	106 (79–142)	11 (4–28)
**Gender**						
Female	298 (269–329)	1000 (772–1000)	293 (258–328)	0	66 (52–81)	13 (6–19)
Male	283 (254–313)	-	298 (262–334)	0	45 (32–58)	3 (0–6)

^1^Per 1000 person-years at risk.

^2^ CI: Confidence Interval.

#### LST reversion and reactivity loss

Of the 1114 individuals who were tested LST positive at enrolment, 61 reverted their LST to negative after one epidemiologic season with a mean decrease in the reaction size of 6.84 mm [6.26–7.34]. All of these 61 subjects were living in the new focus.

Overall, the rate of LST reversion was 56/1000 person-years at risk. It was also higher in persons over 25 years old (p = 0.036) and in females compared to males (p = 0.029).

We observed a loss of LST reactivity in 47 out of 1730 (2.7%) people who had LST1 and LST2, none of them had any comorbidity or used any immunosuppressive drug. As already noted, such reversion was not recorded in the old focus.

The rate of LST reactivity loss was 8/1000 person-years in both foci combined. The higher rates were found in the age groups under 25-year old (p <10^−3^). No statistically significant difference was found in rates of LST reactivity loss between males and females.

### Cutaneous Leishmaniasis cases (symptomatic infection)

Among the 2093 subjects who completed the clinical follow up for active case detection during one year, we identified 60 incident CL cases (new foci: 53 of 1688; 3.1%; old focus: 7 of 405; 1.7%). If one considers the 1730 subjects who had both LST1 and LST2 measures, 50 developed patent CL lesion. The ten other CL cases occurred in the 363 participants (2.8%) who were tested for LST1 and had a clinical follow up, but were not tested for LST2. Only one of these ten incident cases occurred in the old focus.

Microscopic examination of smeared exudates from cutaneous sores, detected *Leishmania* amastigotes in all incident cases (100%). Culture in SLC medium was done in 43 cases out of 60. It led to the growth of promastigotes in 27 samples (62.8%), was negative in 11 (25.6%), and was contaminated in five (11.6%).

**Fig 2:**
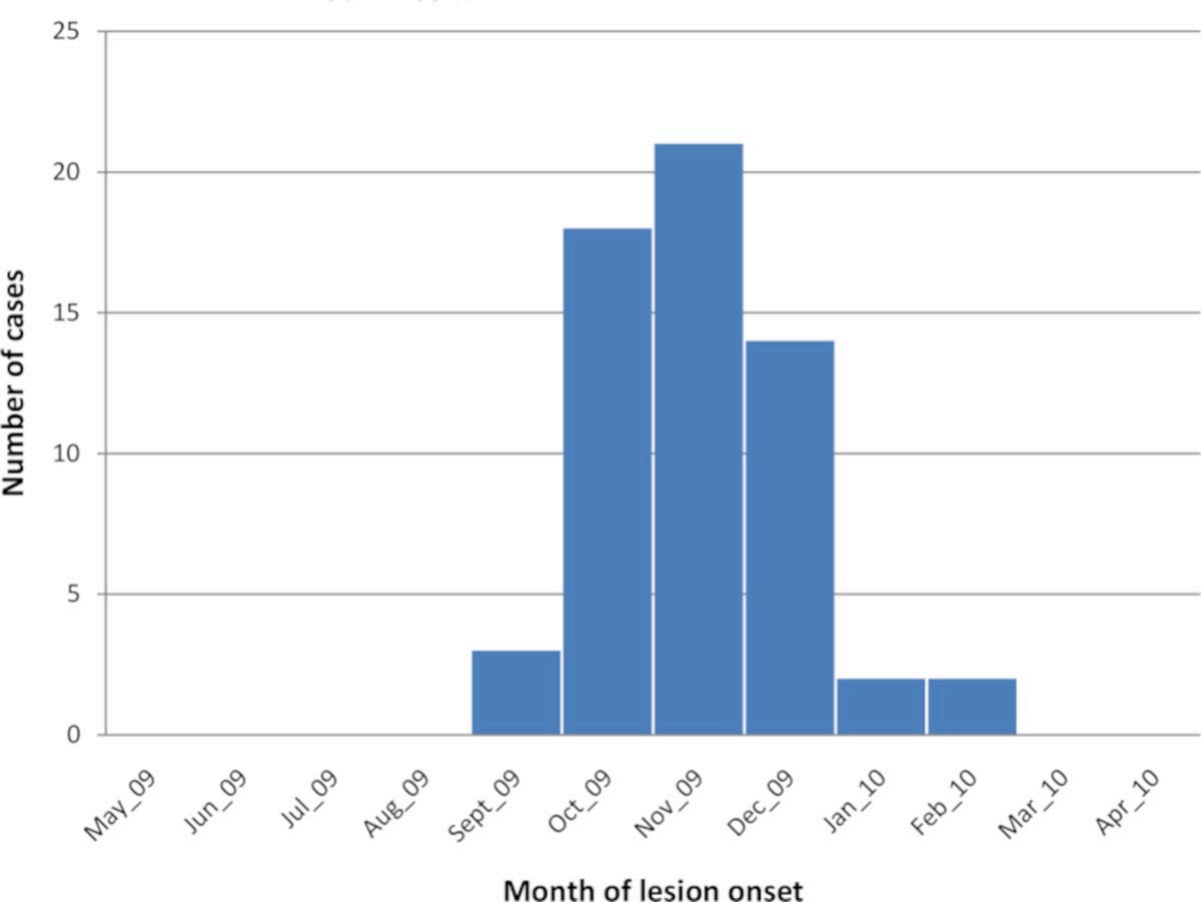
Month of onset of actively detected Cutaneous Leishmaniasis lesions.

Fig 2 illustrates the temporal pattern of lesion onset. Most lesions (88%) appeared between October and December of 2009. Lesions developed at any time from infancy to old age with a mean age of patients at 24.20 ±16.8 (range 6–65 years). A significant association was found between age and CL lesion onset (p≤10^−3^). About 50% of cases occurred in the range 5–15 years. Of the 60 cases of active CL, 25 (42%) were males and 35 (58%) females (p = NS).

**Fig 3:**
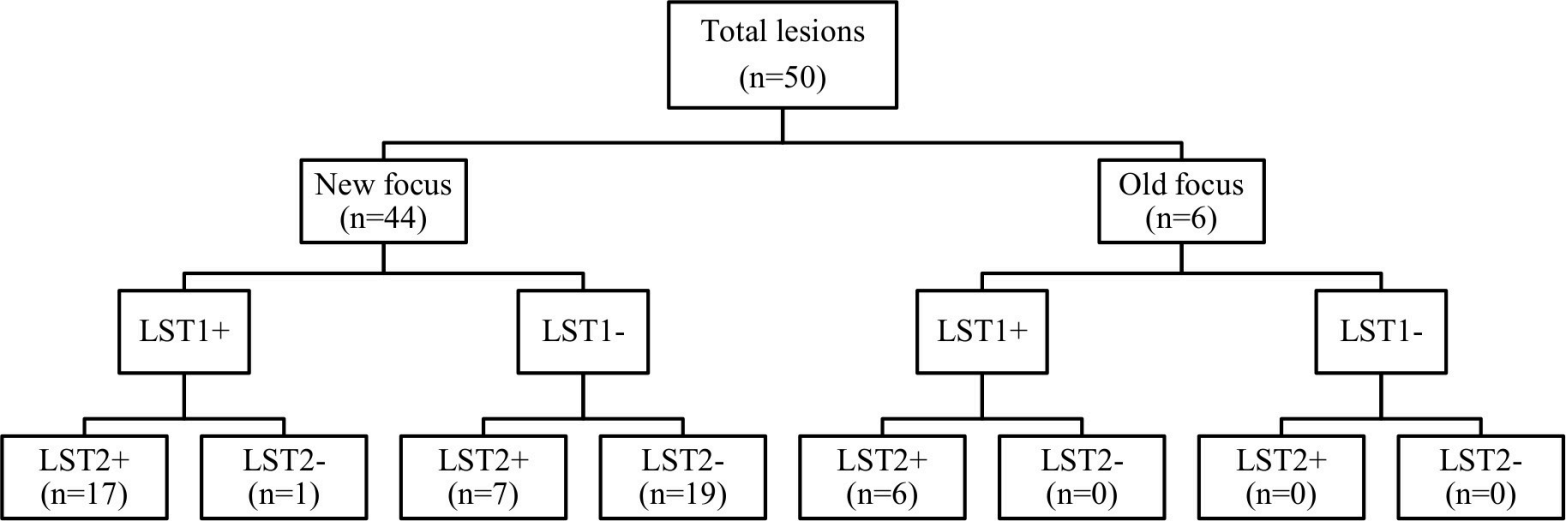
The observed counts of lesions, by focus, according to the Leishmanin Skin Test (LST) positivity before and after one Zoonotic Cutaneous Leishmaniasis emergence season. LST+: Positive Leishmanin skin test, LST-: Negative Leishmanin skin test, n: number of Cutaneous Leishmaniais active lesions. LST1: The first LST performed at baseline: March-May 2009, just before *Leishmania major* transmission season. LST2: The second LST applied to all volunteers on May 2010, just before the next transmission season.

Fig 3 shows the observed results of CL active lesions detected in each study focus after one ZCL season. The 50 CL cases for whom LST1 and LST2 results were available were distributed as follows with regard to changes in the LST reactivity: 7 individuals (14%) converted a negative LST1 to a positive LST2 with a mean increase in size of the LST reaction of 10.5 mm; 23 individuals (46%) had a positive LST1 and a positive LST2 after one year with a mean increase in size of the LST reaction of 2.5 mm. For 19 individuals (38%) LST1 and LST2 were both negative with a 0.7 mm mean increase in the size of LST reaction. Only one patient in this group was diabetic and none of the others had chronic disease or were taking immunosuppressive drugs. Twelve of these cases (63.2%) had one lesion only and all lesions were >3 months old when LST2 was performed. The last CL case (2%), aged 9, had a borderline positive LST1 and a 1.5 mm decrease in LST2 reactivity size compared to LST1. The two latter phenotypes of LST size decrease were only observed in the new (young) focus.

The incidence rate of leishmaniasis during the study period in the two foci combined was 29/1000 person-years at risk (Table 3). The risk of CL case emergence was almost 2.4 times higher in the new focus compared to the old one (p = 0.001). The risk of developing ZCL lesions was also significantly higher in the age group 5-25-year old than in more aged groups. Incidence was greater in males than females, although the difference did not reach statistical significance.

**Table 3:** Incidence rates of Cutaneous Leishmaniasis lesions by focus, age and gender. (Counts reflect weighting).

	N^1^	Person-years	Rate^2^ [95% CI^3^]	RR^4^ [95% CI]	P value
**Focus**					
New	101	3015	34 [27–41]	2.43 [1.37–4.30]	0.001
Old	13	942	14 [6–21]		
**Age (years)**					
[5–25[	69	1971	35 [27–43]	1.55 [1.07–2.24]	0.020
[25–65]	45	1986	23 [16–30]		
**Gender**					
Male	60	1882	32 [24–40]	1.23 [0.85–1.76]	NS^5^
Female	54	2075	26 [19–33]		
**Total**	114	3957	29 [24–34]		

^1^N: weighted number of incident leishmaniasis cases

^2^Per 1000 person-years at risk

^3^CI: Confidence Interval

^4^RR: Risk ratio

^5^NS: Non-significant.

Importantly, the incidence rates of symptomatic infection did not differ significantly according to the results of LST at enrolment (either negative or positive) and this was also true when combining data from both foci, as well as when considering only data from the new focus (Table 4).

**Table 4:** Incidence rates of Cutaneous Leishmaniasis according to baseline Leishmanin Skin Test (LST) positivity (counts reflect weighting).

Baseline immune status	N^1^	Person-years	Rate^2^[95% CI^3^]	RR^4^ [95% CI]
**Both foci combined**				
LST positive	58	2212	26 [19–33]	1
LST negative	56	1745	32 [24–40]	0.92 [0.63–1.36]
**New focus**				
LST positive	45	1283	35 [25–45]	1
LST negative	56	1732	32 [24–40]	1.22 [0.85–1.76]
**Old focus**				
LST positive	13	942	14 [6–21]	-
LST negative	0	0	-	-

^1^N: weighted number of incident leishmaniasis cases

^2^Per 1000 person-years at risk

^3^CI: Confidence Interval

^4^RR: Risk ratio.

## Discussion

This cohort study was conducted in two types of foci endemic for ZCL due to *L major* (an old focus and a more recent one) to better understand over a one year follow up, the natural history and dynamics of infection and the predictive value of LST for overt disease occurrence. We show that the incidence of infection, as assessed by LST conversion rate, was high in the study area, but individuals were at higher risk of developing symptomatic leishmaniasis in the new (young) focus only. Likewise, the rate of LST reversion and reactivity loss was significantly higher in the new focus. Loss of LST reactivity was not noticed in the old focus. Most importantly, the occurrence of overt CL in both foci was irrelevant to the pre-existent LST status.

This study is the first one conducted in North Africa to prospectively estimate the incidence of CL infection at the populational level using a representative sample of exposed individuals. When the present study was implemented in 2009–2010, one focus was categorized as recent/young (i.e. LCZ cases have appeared in this area for less than 5 years), compared to the old focus where LCZ cases were reported since several decades earlier. Even if the status of that focus is no longer young today, the concentric spatial extension of ZCL in Central Tunisia since 1980 [1] continuously creates new foci where the main observations reported in the present study, most likely still hold true. Overall, the incidence rate of newly acquired *Leishmania* infection (defined as the rate of LST conversion) was 295/1000 person-years, of whom 97% exhibited no symptoms as the incidence rate of overt CL was 29/1000 person-years. This figure highlights the fact that in the two study areas, the very large majority of individuals bitten by infected sandflies, will remain asymptomatic. Strictly speaking, we cannot exclude that our survey may have missed some cutaneous sores appearing beyond the end point of the study, either because the inoculation of the parasite by sandfly bite occurred lately during the transmission season, or because of a very long incubation period of the disease or because reactivation of latent parasites as occasionally reported [12]. Indeed, although most CL do have incubation periods that range from two weeks to few months, longer incubation periods extending up to several years have been occasionally reported in Old World CL [13, 14]. However, this bias is likely marginal. Besides, the warming trend observed during the past few decades due to climate change has or may have resulted in the prolongation of the warm season, and thus in the lengthening of the transmission season [15, 16].

Similar results were reported in a prospective study conducted in Colombia which investigated the dynamics of CL infection and clinical manifestations using LST conversion [12]. Study areas were considered to be of low to moderate endemicity. Overall incidence rates infection and disease found were 66/1000 person-years and 4.7/1000 person-years, respectively. Most primary infections (91%) were subclinical [12]. Subclinical infections seem to be more frequent in endemic areas with low-exposure level to *Leishmania* strains [5, 12]. It was proposed that low virulence parasites might confer protection [17]. Unfortunately, we could not compare the rates of asymptomatic infection between old and new foci in our study due to the very low number of incident infections in the old focus. Another longitudinal study on Andean CL in six Peruvian valleys revealed a lower percentage of subclinical infections(14–17%) [4]. One should stress the difficulty to compare between epidemiologic studies conducted in different countries because of differences in transmission rates, circulating *Leishmania* strains, the type of leishmanin used and the characteristics of the study population.

Some studies have suggested that one single administration of LST, is capable by itself, to induce an immune response in the recipient or stimulate a pre-existing immune response to *Leishmania* parasites [18, 19]. A repeated skin testing for diagnostic purposes can therefore hinder the interpretation of LST reaction and may elicit LST conversion to positive in people previously tested negative. As such, it might result in some overestimation of LST conversion rate in our study.

When incidence rates were analysed according to age, there was a higher rate of LST conversion in older people over age 25, while the rate of LST reactivity loss and the risk of symptomatic infection were higher among younger ages. These interesting findings are consistent with many prior reports which indicate that older persons with a longer average duration of stay in endemic areas might have had longer exposure to infective sandfly bites which continuously boost their immune response, compared to younger people [12, 20, 21]. Thus, most (re)-infections in adults remain symptomless and positive LST at this age might persist lifelong.

ZCL control is difficult due to the complexity of transmission cycle and the costliness of effective strategies compared to the limited resources available in developing countries. Hence, vaccine development would be the most cost effective control strategy for CL. So far, attempts to develop such vaccine have failed for several reasons [22, 23]. The validity of the methodology applied in vaccine clinical trials may also be questioned. Indeed, the LST reaction has been used in such trials, not only as a criterion for inclusion of study participants (as LST negative individuals), but also as an outcome indicator of vaccine efficacy [24, 25]. Participants with negative LST were presumed immunologically naïve to the parasite and susceptible to infection; Hence they were included in vaccine trials investigating the immunogenicity and efficacy of vaccine candidates as featured by LST conversion [18, 25, 26]. As discussed above, a sensitization to Leishmania antigens can be induced by a single LST application to individuals living in areas free of Leishmaniasis [18, 19]. This fact, stresses the necessity to re-evaluate the usefulness of LST in clinical vaccine trials against Leishmaniasis.

Our longitudinal study allowed us to evaluate the dynamics of LST reactivity under two different past history of transmission. The individual LST positivity appeared fairly stable over time in old endemic areas where transmission of *Leishmania* was continuing for more than 25 years, whereas in the new focus, a significant LST reversion (to negative) was observed following one single epidemiologic season. Thus, persons living in an old focus who got infected long ago and were continuously exposed to the parasite, are more likely to display a long lasting positive LST reactivity [27] and LST negative individuals are very scarce in such setting. In contrast, individuals with recently acquired infection are more prone to lose their LST positivity over time, accounting for the observed LST fluctuation in recent foci [28] and as corroborated by our findings.

A rapid decay of a weak memory T cells response over the one year follow up long term memory responses are dependent on the persistence of antigenic stimulation by residual parasites. In the mouse model of leishmaniasis, once parasites are eliminated, mice lost their immunity indicating that persistent parasites are important in maintaining a long term cell-mediated immune response [27]. Hence, we may consider that the development in humans of a long term memory T cells could be hampered by a total clearance of parasites after CL healing. In fact, our group has previously reported that no parasite *Leishmania* DNA nor revivable *Leishmania* parasite could be recovered from the cutaneous biopsy of the scar of healed cutaneous leishmaniasis due to *L major* [29]. Such result might suggest that a total clearance of *Leishmania* in ZCL due to *L major* is a possible option, at least in some patients. In these cases particularly, the longevity of LST reactivity will rely on the continuous exposure of the patients to *Leishmania* antigens through repeated biting by infected sand flies as experienced in an old established focus with intense transmission. Possibly, a short history of exposure to infected sandfly bites as in a recent focus may lead to a more or less rapid decay and even a loss of LST reactivity. The lack of stability of the positive LST reaction in new (young) foci may be confusing while selecting candidates for vaccine trials.

As there is evidence that exposure to sandfly saliva may play a protective role [30–32], the addition of sandfly salivary components to anti-leishmanial vaccine have been tried to develop novel vaccine candidates [33]. Recently, our group has confirmed that individuals living in an old endemic focus, repeatedly exposed to *Phlebotomus papatasi* sandfly bites, express higher IgG antibodies to sandfly saliva antigens and IFN-γ levels compared to those living in a new focus [34].

Several studies have looked at the relation between lesions characteristics (such as lesion age, lesion localization and time since healing [35–38]) and LST characteristics. It takes between a few days to two months of disease evolution for LST to become positive. Beyond two months, a low LST reactivity should be regarded as a poor host immune response. However, little attention has been paid to the impact of the disease background of the study area on LST performance. We found that few patients (n = 19) from the new focus remained LST negative despite parasitologically confirmed CL of over 3 months old. Except for one patient with diabetes, these cases had neither associated chronic co-morbidity nor immunosuppressive drugs consumption.

Several hypotheses may account for this unusual phenotype. One possibility is that these patients will develop a positive reaction later than 3 months after lesion appearance. Another possibility is that the cut off value of 5 mm that segregates between the negative and positive LST reactions may not be valid to all individuals. Some CL patients may develop a weak LST reaction of less than 5 mm possibly and this phenotype does not preclude development of a potent T cell immunity to *Leishmania* antigens. When comparing the LST reactivity with in vitro lymphoproliferative responses to *Leishmania* antigens we have observed occasional patients with negative LST (<5mm) with otherwise good lymphoproliferative responses [39]. Similar observations were made after BCG vaccination where some apparently healthy individuals fail to convert their tuberculin skin test which remains persistently negative even after revaccination. As for CL patients, it is interesting to note that the phenotype of negative LST2 was observed only in the new (young focus) which suggests that it may be linked to the short history of exposure.

The present study has yielded different findings than those obtained through previous cohort study performed by our group in Dhiba and Remada in Southern-East of Tunisia [5]. These two areas were prototypic recent foci where *L*. *major* invaded the population and reached epidemic dimensions in 1993, more than 20 years after the discovery of this disease in Central Tunisia [5]. This previous study revealed that individuals with a history of ZCL or with asymptomatic infection (CL scars and/or baseline LST positivity) exhibit resistance to reinfection that increases in proportion to the size of the LST reaction at baseline, independently of the transmission pressure [5]. The discrepancy between this study and the present one might be explained, at least in part, by the different age groups involved in the follow-up. The previous cohort was restricted to school children whereas our present cohort included all age groups. Nevertheless, some of our findings might reflect some peculiarities of the study area and should be reproduced in other ecologic settings in order to be generalized.

We found that a negative LST at time of enrolment was not predictive of a significant increased risk of developing overt CL. The risk of developing a new active lesion was almost similar in LST negative individuals supposed to be susceptible to infection and in LST positive ones supposed to be resistant with the former group displaying a higher increase in mean LST reaction size. Our data support that LST positivity might indicate a sensitisation to *Leishmania* antigens possibly triggered by a previous infection but it does not support that it is a marker of protection against disease recurrence. Hence LST positivity should not be considered as a surrogate marker of protective immunity [20, 40, 41].

In a study reported from Iran [20], 273 volunteers, with positive LST at baseline, were enrolled in an area with a very high incidence of CL and then followed for up to 3-year period. The annual incidence of CL in this group was close to the incidence observed during the same frame time among the general population living in the same endemic area, however the severity of the disease was lower in the study group [20]. Furthermore, a study has revealed that patients with South-American CL due to *Leishmania (Viannia) braziliensis* and a negative LST, had a 3.4 fold higher risk of relapse after successful treatment, compared to CL patients with a positive LST [42]. It was concluded that low LST responses predict relapse after treatment of American CL Patients with negative skin test at the time of diagnosis, are in need for higher doses or longer therapy in order to prevent relapse [42]. The impact of LST status on the severity of the subsequent disease, healing duration and disease recurrence, was beyond the scope of our study and warrants further studies.

In humans, the outcome of infection by *Leishmania* parasites is a result of a complex interplay between the host immune system and the inoculated parasites [12, 43–46]. Although many aspects of vector–pathogen interactions have been successfully deciphered, important questions related to mechanisms of resistance or susceptibility to *Leishmania* in humans, are still poorly understood. Our study stresses the importance of investigating the mechanisms of immunity that efficiently protect people against symptomatic infection, by electively targeting human populations living in old endemic foci where they are continuously exposed to parasites (re) inoculated by infective sandfly bites. This population may have developed on the long term stable mechanisms of resistance against the disease that are not yet fully developed in people living in more recent foci as evidenced by the temporal fluctuations in LST reactivity in such setting. This information will be crucial for the optimal design of anti-*Leishmania* vaccines trials.

## Supporting information

S1 Checklist STROBE Checklist. (DOC)

S1 Table Selected characteristics of study participants who were skin tested before and after the transmission season (Observed counts). (DOCX)

## References

[1] Salah AB, Kamarianakis Y, Chlif S, Alaya NB, Prastacos P. Zoonotic cutaneous leishmaniasis in central Tunisia: spatio–temporal dynamics. International Journal of Epidemiology. 2007;36(5):991–1000. 10.1093/ije/dym125 17591639

[2] Montenegro J. Cutaneous reaction in leishmaniasis. Archives of Dermatology and Syphilology. 1926;13(2):187–94.

[3] Salman SM, Rubeiz NG, Kibbi A-G. Cutaneous leishmaniasis: clinical features and diagnosis. Clinics in dermatology. 1999;17(3):291–6. 10.1016/s0738-081x(99)00047-4 10384868

[4] Davies CR, Llanos-Cuentas E, Pyke S, Dye C. Cutaneous leishmaniasis in the Peruvian Andes: an epidemiological study of infection and immunity. Epidemiology and infection. 1995;114(02):297–318. 7705493 10.1017/s0950268800057964 PMC2271273

[5] Salah AB, Louzir H, Chlif S, Mokni M, Zaâtour A, Raouène M, et al The predictive validity of naturally acquired delayed-type hypersensitivity to leishmanin in resistance to Leishmania major–associated cutaneous leishmaniasis. Journal of Infectious Diseases. 2005;192(11):1981–7. 10.1086/498042 16267771

[6] Sokal JE. Measurement of delayed skin-test responses. Mass Medical Soc; 1975. 10.1056/NEJM197509042931013 1152865

[7] ALIMOHAMMADIAN MH, HAKIMI H, NIKSERESHT M. The P Reparation And Evaluation Of Reference Leishmanin From Leishmania Major For Use In Man For Diagnos Tic And Experimental Purposes. Medical Journal of The Islamic Republic of Iran (MJIRI). 1993;7(1):23–8.

[8] Pourmohammadi B, Motazedian M, Hatam G, Kalantari M, Habibi P, Sarkari B. Comparison of three methods for diagnosis of cutaneous leishmaniasis. Iranian journal of parasitology. 2010;5(4):1 22347259 PMC3279850

[9] Bearman JE, Kleinman H, Glyer VV, Lacroix OM. A study of variability in tuberculin test reading. American Review of Respiratory Disease. 1964;90(6):913–9. 14233795 10.1164/arrd.1964.90.6.913

[10] Sautory O, editor Calmar 2: A new version of the calmar calibration adjustment program. Proceedings of Statistics Canada Symposium; 2003.

[11] Deville J-C, Särndal C-E, Sautory O. Generalized raking procedures in survey sampling. Journal of the American statistical Association. 1993;88(423):1013–20.

[12] Weigle KA, Santrich C, Martinez F, Valderrama L, Saravia NG. Epidemiology of cutaneous leishmaniasis in Colombia: a longitudinal study of the natural history, prevalence, and incidence of infection and clinical manifestations. Journal of Infectious Diseases. 1993;168(3):699–708. 10.1093/infdis/168.3.699 8354912

[13] Clay R. INCUBATION PERIOD OF CUTANEOUS LEISHMANIASIS. The Lancet. 1960;275(7117):230.

[14] Nadim A, Javadian E, Tahvildar-Bidruni G, Ghorbani M. Effectiveness of leishmanization in the control of cutaneous leishmaniasis. Bulletin de la Société de Pathologie Exotique et de ses Filiales. 1983;76(4):377–83. 6354498

[15] De la Rocque S, Rioux J. Influence des changements climatiques sur l’épidémiologie des maladies transmissibles. Bull Soc Path Exo. 2008;101:213–9. 18681214

[16] Watson RT, Albritton DL. Climate change 2001: Synthesis report: Third assessment report of the Intergovernmental Panel on Climate Change: Cambridge University Press; 2001.

[17] Neal R, Reeves A, Peters W. Leishmania infecting man and wild animals in Saudi Arabia 7. Partial protection of mice against Leishmania major by prior infection with L. arabica. Transactions of the Royal Society of Tropical Medicine and Hygiene. 1990;84(2):233–8. 10.1016/0035-9203(90)90267-i 2389313

[18] De Luca P, Mayrink W, Santiago M, Nogueira R, Conceição-Silva F, Melo G, et al Randomized, double-blind, placebo-controlled study on the immunogenicity of the leishmanin skin test. Transactions of the Royal Society of Tropical Medicine and Hygiene. 2003;97(6):709–12. 10.1016/s0035-9203(03)80109-8 16117969

[19] Nascimento MD, Alcântara-Neves NM, Muniz MEB, Nunes SF, Paranhos M, de Carvalho LCP. Induction and modulation of the immune response to Leishmania by Montenegro's skin test. Transactions of the Royal Society of Tropical Medicine and Hygiene. 1993;87(1):91–3. 10.1016/0035-9203(93)90439-w 8465411

[20] Momeni Boroujeni A, Aminjavaheri M, Moshtaghian B, Momeni A, Momeni AZ. Reevaluating leishmanin skin test as a marker for immunity against cutaneous leishmaniasis. International journal of dermatology. 2013;52(7):827–30. 10.1111/j.1365-4632.2012.05850.x 23621513

[21] Silveira FT, Lainson R, Crescente JÂ, De Souza AA, Campos MB, Gomes CM, et al A prospective study on the dynamics of the clinical and immunological evolution of human Leishmania (L.) infantum chagasi infection in the Brazilian Amazon region. Transactions of the Royal Society of Tropical Medicine and Hygiene. 2010;104(8):529–35. 10.1016/j.trstmh.2010.05.002 20538310

[22] Gillespie PM, Beaumier CM, Strych U, Hayward T, Hotez PJ, Bottazzi ME. Status of vaccine research and development of vaccines for leishmaniasis. Vaccine. 2016;34(26):2992–5. 10.1016/j.vaccine.2015.12.071 26973063

[23] Kedzierski L. Leishmaniasis vaccine: where are we today? Journal of global infectious diseases. 2010;2(2):177 10.4103/0974-777X.62881 20606974 PMC2889658

[24] Khalil E, Hassan A, Zijlstra E, Mukhtar M, Ghalib H, Musa B, et al Autoclaved Leishmania major vaccine for prevention of visceral leishmaniasis: a randomised, double-blind, BCG-controlled trial in Sudan. The Lancet. 2000;356(9241):1565–9. 10.1016/s0140-6736(00)03128-7 11075771

[25] Sharifi I, FeKri AR, Aflatonian M-R, Khamesipour A, Nadim A, Mousavi M-RA, et al Randomised vaccine trial of single dose of killed Leishmania major plus BCG against anthroponotic cutaneous leishmaniasis in Bam, Iran. The Lancet. 1998;351(9115):1540–3. 10.1016/S0140-6736(98)09552-X 10326536

[26] De Luca PM, Mayrink W, Pinto JA, Coutinho SG, Santiago MA, Toledo VP, et al A randomized double-blind placebo-controlled trial to evaluate the immunogenicity of a candidate vaccine against American tegumentary leishmaniasis. Acta tropica. 2001;80(3):251–60. 10.1016/s0001-706x(01)00181-4 11700183

[27] Scott P. Immunologic memory in cutaneous leishmaniasis. Cellular microbiology. 2005;7(12):1707–13. 10.1111/j.1462-5822.2005.00626.x 16309457

[28] Weigle KA, Valderrama L, Arias AL, Santrich C, Saravia NG. Leishmanin skin test standardization and evaluation of safety, dose, storage, longevity of reaction and sensitization. The American journal of tropical medicine and hygiene. 1991;44(3):260–71. 10.4269/ajtmh.1991.44.260 2035747

[29] Sghaier RM, Benhnini F, Zaatour A, Attia H, Mkannez G, Bali A, et al, editors. Do scars caused by past history of Leishmania major Ainfection may harbor persistent parasites? BMC proceedings; 2011: Springer.

[30] Andrade BdB, De Oliveira C, Brodskyn CI, Barral A, Barral‐Netto M. Role of sand fly saliva in human and experimental leishmaniasis: current insights. Scandinavian journal of immunology. 2007;66(2‐3):122–7. 10.1111/j.1365-3083.2007.01964.x 17635789

[31] Kamhawi S, Belkaid Y, Modi G, Rowton E, Sacks D. Protection against cutaneous leishmaniasis resulting from bites of uninfected sand flies. Science. 2000;290(5495):1351–4. 10.1126/science.290.5495.1351 11082061

[32] Teixeira C, Gomes R, Oliveira F, Meneses C, Gilmore DC, Elnaiem D-EA, et al Characterization of the early inflammatory infiltrate at the feeding site of infected sand flies in mice protected from vector-transmitted Leishmania major by exposure to uninfected bites. Plos neglected tropical diseases. 2014;8(4):e2781 10.1371/journal.pntd.0002781 24762408 PMC3998922

[33] Oliveira F, Rowton E, Aslan H, Gomes R, Castrovinci PA, Alvarenga PH, et al A sand fly salivary protein vaccine shows efficacy against vector-transmitted cutaneous leishmaniasis in nonhuman primates. Science translational medicine. 2015;7(290):290ra90–ra90. 10.1126/scitranslmed.aaa3043 26041707

[34] Kammoun-Rebai W, Bahi-Jaber N, Naouar I, Toumi A, Salah AB, Louzir H, et al Human cellular and humoral immune responses to Phlebotomus papatasi salivary gland antigens in endemic areas differing in prevalence of Leishmania major infection. Plos neglected tropical diseases. 2017;11(10):e0005905 10.1371/journal.pntd.0005905 29023574 PMC5638224

[35] Antonio LdF, Fagundes A, Oliveira RVC, Pinto PG, Bedoya-Pacheco SJ, Vasconcellos ÉdCF, et al Montenegro skin test and age of skin lesion as predictors of treatment failure in cutaneous leishmaniasis. Revista do Instituto de Medicina Tropical de São Paulo. 2014;56(5):375–80. 10.1590/s0036-46652014000500002 25229216 PMC4172107

[36] Boggild AK, Ramos AP, Espinosa D, Valencia BM, Veland N, Miranda-Verastegui C, et al Clinical and demographic stratification of test performance: a pooled analysis of five laboratory diagnostic methods for American cutaneous leishmaniasis. The American journal of tropical medicine and hygiene. 2010;83(2):345–50. 10.4269/ajtmh.2010.09-0414 20682880 PMC2911183

[37] Krolewiecki AJ, Almazan MC, Quipildor M, Juarez M, Gil JF, Espinosa M, et al Reappraisal of Leishmanin Skin Test (LST) in the management of American Cutaneous Leishmaniasis: A retrospective analysis from a reference center in Argentina. PLoS neglected tropical diseases. 2017;11(10):e0005980 10.1371/journal.pntd.0005980 28981507 PMC5645152

[38] Manzur A. Sensitivity of leishmanin skin test in patients of acute cutaneous leishmaniasis. Dermatology online journal. 2006;12(4). 17083857

[39] Sassi A, Louzir H, Ben Salah A, Mokni M, Ben Osman A. Dellag i K. Leishmanin skin test lymphoproliferative responses an d cytokine production after symptomatic or asymptomatic Leishmania major infection in Tunisia. Clin Exp Immunol. 1999;116:127–32. 10.1046/j.1365-2249.1999.00844.x 10209516 PMC1905205

[40] Diniz JLCP, Costa MOdR, Gonçalves DU. Mucocutaneous Leishmaniasis: clinical markers in presumptive diagnosis. Brazilian journal of otorhinolaryngology. 2011;77(3):380–4. 10.1590/s1808-86942011000300018 21739015 PMC9443764

[41] Ponte CB, Souza NC, Cavalcante MN, Barral AMP, Aquino DMCd, Caldas AdJM. Risk factors for Leishmania chagasi infection in an endemic area in Raposa, State of Maranhão, Brazil. Revista da Sociedade Brasileira de Medicina Tropical. 2011;44(6):712–21. 10.1590/s0037-86822011005000059 22094705

[42] Passos V, Barreto SM, Romanha AJ, Krettli AU, Volpini ÂC, Costa MFF. American cutaneous leishmaniasis: use of a skin test as a predictor of relapse after treatment. Bulletin of the World Health Organization. 2000;78:968–74. 10994280 PMC2560824

[43] Aebischer T, Moody SF, Handman E. Persistence of virulent Leishmania major in murine cutaneous leishmaniasis: a possible hazard for the host. Infection and immunity. 1993;61(1):220–6. 10.1128/IAI.61.1.220-226.1993 8093358 PMC302708

[44] Louzir H, Melby PC, Salah AB, Marrakchi H, Aoun K, Ismail RB, et al Immunologic determinants of disease evolution in localized cutaneous leishmaniasis due to Leishmania major. Journal of Infectious Diseases. 1998;177(6):1687–95. 10.1086/515297 9607850

[45] Machado PR, Carvalho AM, Machado GU, Dantas ML, Arruda S. Development of cutaneous leishmaniasis after Leishmania skin test. Case reports in medicine. 2011;2011. 10.1155/2011/631079 PMC3227237 22162702

[46] Reithinger R, Dujardin J-C, Louzir H, Pirmez C, Alexander B, Brooker S. Cutaneous leishmaniasis. The Lancet infectious diseases. 2007;7(9):581–96. 10.1016/S1473-3099(07)70209-8 17714672

