# Supplementary material for: A prospective cohort study of Cutaneous Leishmaniasis due to Leishmania major: Dynamics of the Leishmanin skin test and its predictive value for protection against infection and disease
Source: PLoS Negl Trop Dis. 2020 Aug 25;14(8):e0008550. doi: 10.1371/journal.pntd.0008550 (PMC7473511; doi:10.1371/journal.pntd.0008550)
Supplement: S1 Checklist — (DOC) [file pntd.0008550.s001.doc]

**S1 Checklist: STROBE Checklist**

|  | Item No | Manuscript section and paragraph number |
| --- | --- | --- |
| **Title and abstract** | 1 | (*a*) (Title ) |
|  |  | (*b*) (Abstract) |
| Introduction | | |
| Background/rationale | 2 | (Introduction, Paragraph 1, 2) |
| Objectives | 3 | (Introduction, Paragraph 3) |
| Methods | | |
| Study design | 4 | (Methods, Section field word ) |
| Setting | 5 | (Methods, Sections field word & Enrolment and follow-up of participants) |
| Participants | 6 | (Methods, Section: Enrolment and follow-up of participants ) |
| Variables | 7 | (Methods, Section Outcomes definitions and analysis plan) |
| Data sources/ measurement | 8 | (Methods, Section Outcomes definitions and analysis plan) |
| Bias | 9 | (Methods, Section: Enrolment and follow-up of participants , last sentence ) |
| Study size | 10 | (Methods, Section: Enrolment and follow-up of participants , Paragraph 2:The study size was previously described in more details see ref 4) |
| Quantitative variables | 11 | (Methods, Section Outcomes definitions and analysis plan) |
| Statistical methods | 12 | (Methods, Section Outcomes definitions and analysis plan) |
| Results | | |
| Participants | 13 | (a) (Results, Paragraph 1) |
|  |  | (b) (Results, Paragraph 1) |
|  |  | (c) (Results, figure 2) |
| Descriptive data | 14 | (a) (Results, Table 1) |
|  |  | (b) (Results, Paragraph 3) |
|  |  | (c) (Results, Section: Leishmaniasis cases, paragraph 1) |
| Outcome data | 15 | (Results, figure 2) |
| Main results | 16 | (Results, table 2) |
| Other analyses | 17 | (Results, Section: Leishmaniasis cases, Table 3, 4 ) |
| Discussion | | |
| Key results | 18 | (Discussion, Paragraph 1) |
| Limitations | 19 | (Discussion, Paragraph 2) |
| Interpretation | 20 | (Discussion, Paragraph 2,3,4,5) |
| Generalisability | 21 | (Discussion, Paragraph 7) |
| Other information | | |
| Funding | 22 | (the source of funding and its role were mentioned in the reserved section of the online submission system) |
