# Supplementary material for: A prospective cohort study of Cutaneous Leishmaniasis due to Leishmania major: Dynamics of the Leishmanin skin test and its predictive value for protection against infection and disease
Source: PLoS Negl Trop Dis. 2020 Aug 25;14(8):e0008550. doi: 10.1371/journal.pntd.0008550 (PMC7473511; doi:10.1371/journal.pntd.0008550)
Supplement: S1 Table — (DOCX) [file pntd.0008550.s002.docx]

**S1 Table. Selected characteristics of study participants who were skin tested before and after the transmission season (Observed counts).**

|  | **Old focus**  **(n= 335)** | **New focus**  **(n= 1395)** | **Both foci**  **(n=1730)** | **P value** |
| --- | --- | --- | --- | --- |
| **Median Age (IQR^1^) (yrs)** | 19 (13-47) | 27 (12-44) | 26 (13-44) | NS^2^ |
| **Females %** | 63 | 67 | 66 | NS |
| **Education %** |  |  |  | NS |
| Under school age | 2 | 4 | 3 |  |
| Illiterate | 25 | 22 | 23 |  |
| Primary | 47 | 54 | 53 |  |
| Secondary | 24 | 19 | 20 |  |
| University | 2 | 1 | 1 |  |
| **Farming occupation %** | 1 | 3 | 2 | NS |
| **History of ZCL/Scars %** | 34 | 18 | 21 | <10^-3^ |

^1^IQR: Interquartile range.

^2^NS: Non significant.
